# Supplementary material for: Higher Serum Total Cholesterol to High-Density Lipoprotein Cholesterol Ratio Is Associated with Increased Mortality among Incident Peritoneal Dialysis Patients
Source: Nutrients. 2021 Dec 29;14(1):144. doi: 10.3390/nu14010144 (PMC8746736; doi:10.3390/nu14010144)
Supplement: Supplementary file 1 [file nutrients-14-00144-s001.zip › nutrients-1475510-supplementary.pdf]

Supplemental materials

Supplement Table S1. Relationship between serum TC/HDL-C ratio and mortality (Reference group; Quintile 1)

Supplement Table S2. Relationship between serum LDL-C and mortality

Table S1. Relationship between serum TC/HDL-C ratio and mortality (Reference group; Quintile 1)

	Quintile 2		Quintile 3		Quintile 4		Quintile 5	
	**HR (95%CI)**	**p-value**	**HR (95% CI)**	**p-value**	**HR(95% CI)**	**p-value**	**HR(95%CI)**	**p-value**
Model 1	1.10 (0.69-1.75)	0.689	0.79 (0.48-1.29)	0.347	1.08 (0.68-1.71)	0.759	1.37 (0.88-2.13)	0.163
Model 2	1.04 (0.65-1.67)	0.862	0.85 (0.51-1.42)	0.535	1.11 (0.69-1.79)	0.670	1.41 (0.88-2.25)	0.153
Model 3	1.02 (0.63-1.64)	0.948	0.79 (0.47-1.32)	0.366	1.15 (0.71-1.86)	0.579	1.34 (0.83-2.15)	0.232
Model 4	1.02 (0.63-1.64)	0.943	0.79 (0.47-1.33)	0.374	1.15 (0.71-1.86)	0.582	1.34 (0.83-2.15)	0.233

HR, hazard ratio. Reference group was Quintile 1. Model 1: Unadjusted. Model 2: Model 1 plus age, sex and body mass index. Model 3: Model 2 plus laboratory data and MCCI. Model 4: Model 3 plus 24 hour urine volume.

Table S2. Relationship between serum LDL-C and mortality

	Quintile 1		Quintile 2		Quintile 4		Quintile 5	
	**HR (95% CI)**	**p-value**	**HR (95% CI)**	**p-value**	**HR (95% CI)**	**p-value**	**HR (95% CI)**	**p-value**
Model 1	1.38 (0.86-2.20)	0.182	1.24 (0.77-2.00)	0.369	1.10 (0.68-1.77)	0.708	1.34 (0.85-2.12)	0.206
Model 2	1.26 (0.79-2.03)	0.331	1.32 (0.82-2.12)	0.262	1.15 (0.71-1.86)	0.570	1.28 (0.81-2.04)	0.287
Model 3	1.05 (0.65-1.70)	0.847	1.27 (0.78-2.05)	0.338	1.13 (0.69-1.83)	0.628	1.02 (0.63-1.65)	0.950
Model 4	1.05 (0.64-1.70)	0.856	1.26 (0.77-2.05)	0.357	1.12 (0.69-1.83)	0.644	1.02 (0.63-1.65)	0.950

HR, hazard ratio. Reference group was Quintile 3. Model 1: Unadjusted. Model 2: Model 1 plus age, sex and body mass index. Model 3: Model 2 plus laboratory data and MCCI. Model 4: Model 3 plus 24 hour urine volume.
